# Upward and downward comparisons across monetary and status domains

**DOI:** 10.1002/hbm.25148

**Published:** 2020-07-23

**Authors:** Zachary A. Yaple, Rongjun Yu

**Affiliations:** ^1^ Department of Psychology National University of Singapore Singapore; ^2^ NUS Graduate School for Integrative Sciences and Engineering, National University of Singapore Singapore

**Keywords:** reward, social comparison, social status

## Abstract

The ability to accurately infer one's place with respect to others is crucial for social interactions. Individuals tend to evaluate their own actions and outcomes by comparing themselves to others in either an upward or downward direction. We performed two fMRI meta‐analyses on monetary (*n* = 39; 1,231 participants) and status (*n* = 23; 572 participants) social comparisons to examine how domain and the direction of comparison can modulate neural correlates of social hierarchy. Overall, both status and monetary downward comparisons activated regions associated with reward processing (striatum) while upward comparisons yielded loss‐related activity. These findings provide partial support for the common currency hypothesis in that downward and upward comparisons from both monetary and status domains resemble gains and losses, respectively. Furthermore, status upward and monetary downward comparisons revealed concordant orbitofrontal cortical activity, an area associated with evaluating the value of goals and decisions implicated in both lesion and empirical fMRI studies investigating social hierarchy. These findings may offer new insight into how people relate to individuals with higher social status and how these social comparisons deviate across monetary and social status domains.

## Graphical abstract

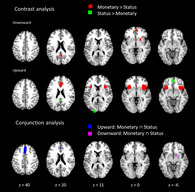

## 1 INTRODUCTION

One of the cornerstones of social cognition is the ability to compare oneself to others in social contexts (Mussweiler, 2003). Social comparisons occur on a day‐to‐day basis as a means to gain accurate self‐evaluations in a competitive hierarchical society (Festinger, 1954). There has been growing interest in exploring the neurological underpinnings of social comparisons using functional magnetic resonance imaging (fMRI). For example, neurobiological reviews on social comparisons have suggested a possible role of the reward system and the frontal–parietal system (Chiao, 2010; Kedia, Mussweiler, & Linden, 2014). Convergent evidence from multiple neuroimaging studies on social hierarchy reveal a network of brain regions associated with social status (i.e., ventral striatum, regions of the prefrontal cortex, inferior parietal lobe, and portions of the occipital lobe; Chiao et al., 2008, 2009; Zink et al., 2008; Freeman, Rule, Adams Jr, & Ambady, 2009; Marsh, Blair, Jones, Soliman, & Blair, 2009; Bault, Joffily, Rustichini, & Coricelli, 2011; see Chiao, 2010). The inferior parietal lobe is traditionally viewed to reflect numerical magnitude (Brannon, 2006; Cohen‐Kadosh, Lammertyn, & Izard, 2008; Dehaene, Piazza, Pinel, & Cohen, 2003), thus it has been suggested that this region ascribes social distance of individuals such that activity within sub‐regions of inferior parietal lobe may depend on the relative distance to one's own level (Chiao, 2010; Chiao et al., 2009). Convincing evidence for the prefrontal cortex in social comparisons derives from patients with brain lesions to the ventrolateral, ventromedial and dorsolateral prefrontal cortex, demonstrating impaired attribution to social status (Karafin, Tranel, & Adolphs, 2004; Mah, Arnold, & Grafman, 2004). Recent developments in computational neuroscience that combine neural data with reinforcement learning algorithms have shown that the medial prefrontal cortex selectively mediates the updating of knowledge about one's own rank with respect to others (Kumaran, Banino, Blundell, Hassabis, & Dayan, 2016). Along with the prefrontal cortex, the ventral striatum is said to have a specific role in downward comparisons during which a person with higher status or larger gains is compared with an opponent (Dvash, Gilam, Ben‐Ze'ev, Hendler, & Shamay‐Tsoory, 2010; Cikara, Botvinick, & Fiske, 2011; Meshi, Morawetz, & Heekeren, 2013; Lindner et al., 2015; also see Luo, Eickhoff, Hétu, & Feng, 2018 for meta‐analysis). This area has been hypothesized to reflect reward‐related activation analogous to the striatal‐cortical encoding of reward (Lindner et al., 2015; Luo et al., 2018).

A recent fMRI meta‐analysis on social comparisons supports the idea that social comparisons are associated with activity in the reward network (Luo et al., 2018). The authors revealed reward‐related brain regions (bilateral ventral striatum) associated with downward comparisons (i.e., comparing self with a lower level individual) and loss‐related brain regions, including the bilateral insula and anterior cingulate cortex (ACC) associated with upward comparisons (i.e., comparing self with a higher level individual). The authors infer their data to support the “common‐currency” hypothesis which declares that neural representations of social comparisons in downward and upward directions may resemble neural processing for monetary gains and losses, respectively. However, this recent meta‐analysis of upward and downward comparisons aggregated monetary as well as social status comparisons, focusing on upward and downward comparisons in a more general sense (Luo et al., 2018). Social comparisons can be generated by either comparing monetary outcomes between individuals or by comparing individuals' social status. For example, the Ultimatum game captures events in which one compares oneself with another's monetary gains and losses (see Gabay, Radua, Kempton, & Mehta, 2014; Feng, Luo, & Krueger, 2015 for meta‐analyses). Actively tracking whether one receives the reward he/she deserves is crucial for reward distribution. Processes associated with social status have been assessed by displaying dominant or submissive characteristics such as personality traits (Beer & Hughes, 2010; Moore III, Merchant, Kahn, & Pfeifer, 2014), bodily postures (Freeman et al., 2009; Marsh et al., 2009), facial expressions (Chiao, 2010; Chiao et al., 2008, 2009), or when participants are passively viewing players with high and low rank with respect to their own (Kishida, Yang, Quartz, Quartz, & Montague, 2012; Kumaran, Melo, & Duzel, 2012). Specifically, social status ranking may depend on financial status, occupation status (Cloutier, Ambady, Meagher, & Gabrieli, 2012; Cloutier & Gyurovski, 2013, 2014), or perhaps position in a hierarchy in a competitive setting (Cikara et al., 2011; Lindner et al., 2015). Hence, the domain specific neural representation of social comparison has not yet been systematically examined. Secondly, monetary comparisons offering rewards and penalties may confound the results of the previous meta‐analysis since monetary incentives are very likely to yield gain‐ and loss‐related regions (e.g., *striatum* and cingulate cortex; Chiao, 2010; Kedia et al., 2014). Hence, it is unclear whether their findings reflect social comparisons or merely the registration of different amounts of reward in the brain. Therefore, whether the common‐currency hypothesis is supported by social comparison theory across domains other than monetary social comparisons remains to be seen.

To overcome these limitations, we attempt to perform separate fMRI meta‐analyses on monetary and status social comparisons to determine the concordance of functional brain activation across studies and thereby re‐testing the common‐currency hypothesis. The separation of these types of social comparisons will allow us to test the common currency hypothesis for both monetary and status domains, independently. We will examine how upward and downward social status and monetary comparisons differ in functional neural activity, and how these comparisons overlap in the brain by performing additional contrast and conjunction analyses, respectively. We adopt the hypothesis put forth by Luo et al. (2018) in that monetary and social status comparisons will yield brain activity analogous to gains and losses (e.g., *striatum* and cingulate). For both status and monetary domains, we expect to find more reward‐related brain activity (e.g., striatum) for downward comparisons, while upward comparisons will yield regions associated with losses (e.g., cingulate/insula). From our results, we aim to shed light on how social comparison engages similar brain regions across status and monetary domains.

## 2 METHODS

Two independent searches were performed for separate meta‐analyses on social status comparisons and monetary social comparisons. To compile studies measuring social status we performed several searches using the key terms: “social hierarchy” AND fMRI, schadenfreude AND fMRI, and “social comparison” AND fMRI into web of knowledge database (http://www.webofknowledge.com) on May 31st, 2018. These three searches yielded a total of 37 articles. Three articles were duplicated across the three searches, and the remaining 34 articles were screen for eligibility. An additional search in review articles yielded 10 more studies, totaling to 44 articles. Our exclusion criteria excluded contrasts that: (a) did not report foci in Talairach or Montreal Neurological Institute (MNI) standard stereotactic coordinate space, (b) did not use whole‐brain fMRI analysis, (c) did not report healthy human participants or (d) did not capture a comparison of social status. Both researchers reached agreement on which experiments meet the general and specific inclusion and exclusion criteria. In total, 26 studies were deemed eligible for inclusion.

The second search of articles relating to monetary social comparisons was extracted from the prior meta‐analysis on social comparisons (Luo et al., 2018). The results of the monetary social comparison were equivalent to the results obtained by Luo and colleagues. Of the 48 original articles, four articles were included in the meta‐analysis of social status, and 38 articles were used to depict monetary social comparisons.

**FIGURE 1:**
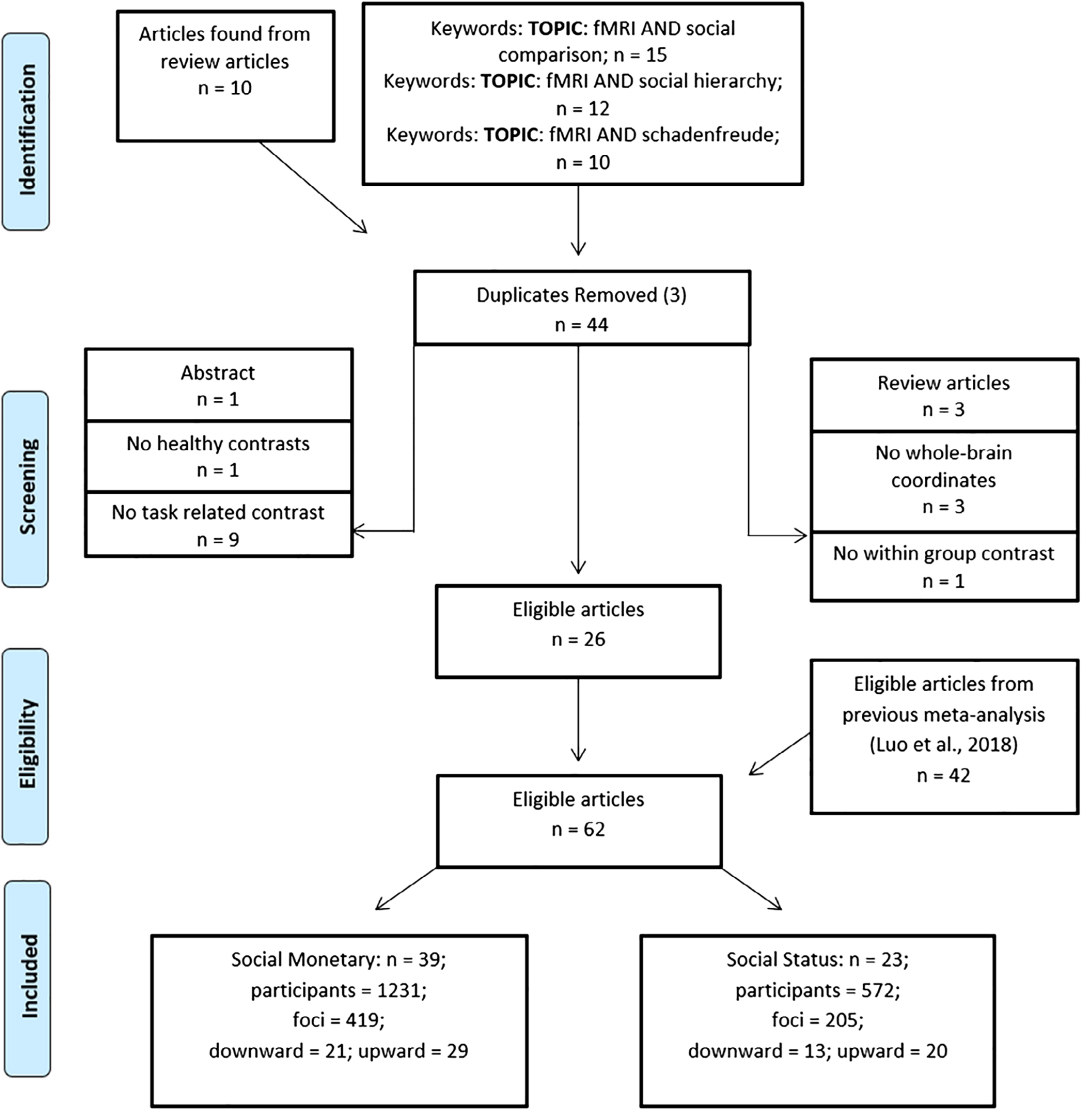
PRISMA flowchart for eligible articles

In total, 62 articles were compiled and divided into upward and downward social status and monetary comparisons (see Figure 1 for more details). Foci between the social status and monetary comparisons were mutually exclusive, although many articles may have reported both upward and downward comparisons. Upward comparisons were selected based on instances in which the player: rated an opponent as inferior, was greater in rank, had achieved larger outcomes than the opponent, or had viewed/judged a face/body posture. Downward comparisons were defined as instances in which the player: rated an opponent as superior, was lower in rank, had achieved less outcomes than the opponent, or had viewed a dominant face/body posture. Social comparison theory was adopted to define operational definitions ‘upward’ and ‘downward’ comparisons. Using this context, we included articles in which participants judged faces/bodily posture as dominant or submissive, as has been done in an earlier study (Zell & Balcetis, 2012). Interestingly, this study confirmed that judgment of faces equivocates to other domains. Tables 1 and 2 display all eligible studies, including stimuli type (monetary or status) and direction of comparison (upward, downward, both).

**TABLE 1:** Information on source datasets included in the meta‐analysis for monetary comparisons

Article	*n*	Mean age (SD)	Male	Foci	Direction	Task type
Assaf et al., 2009 ^b^	19	32.3 (10.4)	10	12	Upward	Gambling
Baumgartner, Knoch, Hotz, Eisenegger, & Fehr, 2011 ^a^ ^,^ ^b^	32	21.6 (2.2)	32	21	Both	Ultimatum game
Civai, Crescentini, Rustichini, & Rumiati, 2012 ^b^	19	NA	7	4	Upward	Ultimatum game
Delgado, Schotter, Ozbay, & Phelps, 2008 ^b^	17	23.77 (3.38)	8	5	Downward	Bidding/lottery
Du et al., 2013 ^b^	19	21.2	19	12	Downward	Dot detection
Dvash et al., 2010 ^a^ ^,^ ^b^	39	24.45 (2.91)	17	19	Downward	Game‐of‐chance
Fareri & Delgado, 2014 ^b^	18	20.4 (2.15)	10	8	Downward	Card‐guessing
Farmer, Apps, & Tsakiris, 2016 ^b^	18	21.1 (2.4)	4	6	Upward	Ultimatum game
Fatfouta, Meshi, Merkl, & Heekeren, 2018 ^a^ ^,^ ^b^	23	24.35 (3.8)	15	18	Upward	Ultimatum game
Fliessbach et al., 2007 ^b^	33	27.4 (4.8)	33	9	Downward	Estimation
Fliessbach et al., 2012 ^b^	64	27.5	32	1	Upward	Dot detection
Gospic et al., 2011 ^b^	17	NA	5	4	Upward	Ultimatum game
Gradin et al., 2015 ^b^	25	25.44 (5.02)	8	10	Upward	Ultimatum game
Guo et al., 2014 ^b^	18	21.06 (2.1)	5	5	Upward	Ball guessing
Güroğlu, van den Bos, van Dijk, Rombouts, & Crone, 2011 ^a^ ^,^ ^b^	68	14.65 (3.97)	36	9	Upward	Ultimatum game
Harlé & Sanfey, 2012 ^b^	38	43.25	15	15	Both	Ultimatum game
Haruno, Kimura, & Frith, 2014 ^b^	59	21.5	26	4	Upward	Ultimatum game
Haruno & Frith, 2010 ^a^ ^,^ ^b^	52	24 (2.3)	24	13	Both	Number recall
Hertz et al., 2017 ^a^ ^,^ ^b^	32	24.24	18	2	Downward	Advice giving
Kang, Lee, Choi, & Kim, 2013 ^b^	22	42.59	0	7	Downward	Game‐of‐chance
Kätsyri, Hari, Ravaja, & Nummenmaa, 2013 ^a^ ^,^ ^b^	17	24.8	17	15	Both	Social
Kirk, Downar, & Montague, 2011 ^b^	40	36.8 (10.1)	19	11	Upward	Ultimatum game
Kirk et al., 2016 ^b^	50	NA	24	11	Upward	Ultimatum game
Lamichhane, Adhikari, Brosnan, & Dhamala, 2014 ^b^	18	25.2 (6.2)	10	3	Upward	Ultimatum game
Mobbs et al., 2013 ^b^	15	25 (7.3)	8	1	Downward	Input foraging
Morawetz, Kirilina, Baudewig, & Heekeren, 2014 ^b^	28	25 (4.24)	17	5	Downward	Dice roll
Roalf, 2010 ^a^ ^,^ ^b^	27	49.81	13	10	Both	Ultimatum game
Servaas et al., 2015 ^a^ ^,^ ^b^	114	~20.8	0	36	Both	Ultimatum game
Steinbeis & Singer, 2014 ^a^ ^,^ ^b^	20	26.01 (2.34)	10	13	Both	Gambling
van den Bos, Talwar, & McClure, 2013 ^a^ ^,^ ^b^	22	~28.56	11	6	Both	Bidding
Verdejo‐Garcia, Verdejo‐Román, Albein‐Urios, Martínez‐González, & Soriano‐Mas, 2017 ^b^	19	30.84	18	4	Upward	Ultimatum game
Votinov, Pripfl, Windischberger, Sailer, & Lamm, 2015 ^a^ ^,^ ^b^	69	23.8 (5.4)	31	65	Downward	MID
White, Brislin, Meffert, Sinclair, & Blair, 2013 ^b^	20	14.15 (2.29)	13	9	Upward	Ultimatum game
White, Brislin, Sinclair, & Blair, 2014 ^a^ ^,^ ^b^	21	28.1 (8.1)	12	10	Both	Ultimatum game
Wu et al., 2014 ^a^ ^,^ ^b^	18	21.6 (1.8)	5	9	Both	Ultimatum game
Wu, Zang, Yuan, & Tian, 2015 ^b^	27	~22.31	6	1	Upward	Ultimatum game
Zheng et al., 2015	25	21.44 (3.38)	7	15	Upward	Ultimatum game
Zheng et al., 2017 ^b^	21	22.8 (1.4)	9	1	Upward	Ultimatum game
Zhou, Wang, Rao, Yang, & Li, 2014 ^a^ ^,^ ^b^	28	25.07 (3.35)	13	10	Both	Ultimatum game

Abbreviations: n, sample size; NA, not available; MID, monetary incentive delay task.

a More than one contrast.

b Eligible studies adopted from Luo et al. (2018).

**TABLE 2:** Information on source datasets included in the meta‐analysis for status comparisons

Article	*n*	Mean age (SD)	Male	Foci	Direction	Task type
Beer & Hughes, 2010	20	20.7 (1.9)	11	8	Upward	Traits
Chester et al., 2013 ^b^	23	18.78 (0.8)	11	7	Upward	Social
Chiao et al., 2008 ^b^	7	NA	7	10	Both	Faces
Cikara et al., 2011 ^b^	18	23.1	15	12	Both	Rank
Cloutier et al., 2012 ^b^	19	24.2	8	13	Both	Social
Cloutier & Gyurovski, 2013 ^b^	13	~23.8	13	20	Both	Social
Cloutier & Gyurovski, 2014 ^b^	20	24.3 (3.9)	20	17	Both	Social
Feng et al., 2016 ^b^	22	22.23 (1.85)	11	16	Both	Social
Freeman et al., 2009	34	NA	16	5	Upward	Faces
Harris & Fiske, 2007 ^b^	18	20	10	6	Both	Social
Hu et al., 2016 ^b^ ^,^ ^c^	23	21.22 (1.73)	10	5	Upward	Social
Kishida et al., 2012 ^b^ ^,^ ^c^	27	25.1 (0.7)	14	4	Both	Rank
Kumaran et al., 2012	25	19–31^a^	14	4	Upward	Rank
Kumaran et al., 2016	30	19–29^a^	12	2	Upward	Rank
Le Bouc & Pessiglione, 2013 ^c^	32	24.7 (0.9)	20	2	Upward	Social
Ligneul, Obeso, Ruff, & Dreher, 2016	28	22	28	2	Upward	Rank
Ligneul, Girard, & Dreher, 2017	28	22.4 (2.8)	28	2	Upward	Rank
Lindner et al., 2015 ^b^	30	23.93 (5.16)	18	23	Both	Rank
Ly, Haynes, Barter, Weinberger, & Zink, 2011	23	33.41 (6.56)	11	1	Downward	Social
Meshi et al., 2013	31	23.1 (3.2)	14	4	Downward	Social
Op de Macks et al., 2017 ^b^ ^,^ ^c^	58	12.4 (0.92)	0	6	Downward	Social
Takahashi et al., 2009 ^b^	19	22.1 (1.4)	10	7	Both	Traits
Zink et al., 2008 ^b^	24	27.6 (5.1)	12	29	Upward	Rank

Abbreviations: n, sample size; NA, not available.

a Only age range reported.

b More than one contrast.

^c^ Eligible studies adopted from Luo et al. (2018).

### 2.1 Software and analysis

We performed co‐ordinate‐based meta‐analyses by using effect‐size Seed‐based d Mapping (ES‐SDM) software from the SDM project (http://www.sdmproject.com). We used this meta‐analysis toolbox rather than the conventional method (i.e., activation likelihood estimation; Eickhoff, Bzdok, Laird, Kurth, & Fox, 2012) because it offers an improved approach by comparing the standardized volume differences between conditions (as opposed to the raw differences), which accounts for small sample size bias (Radua & Mataix‐Cols, 2012). This method is based on the activation likelihood estimation method and increases statistical power by combining statistical parametric *t*‐maps with peak coordinates of clusters from multiple studies (see Radua & Mataix‐Cols, 2012 for more details). Effect‐size maps are created from the reported *t*‐values. The full width at half maximum (FWHM) in SDM was set at the default (20 mm) to control for false positives (see Radua & Mataix‐Cols, 2012). Statistical maps were then thresholded at *p* = .0005 to control for family‐wise error rate.

### 2.2 Sensitivity analyses

Test–retest reliability was assessed by performing jackknife sensitivity analysis; thresholded at *p* = .0005. Jackknife sensitivity analysis is a linear bootstrapping sampling technique which repeats the meta‐analysis as many times as the number of studies that have been included, removing one study per analysis. If an area remains significant in all or most (>80%) of the combinations of studies, it is considered reliable (Radua & Mataix‐Cols, 2009). SDM values were overlaid onto an anatomical template normalized to MNI space using Mango image viewer software (http://rii.uthscsa.edu/mango/mango.html).

## 3 RESULTS

### 3.1 Eligible studies

Twenty‐three articles were compiled to create a stereotaxic map of social status comparisons. Ten articles reported foci on social status instructing participants to view and rate profiles of inferior or superior real or computer simulated players (Chester et al., 2013; Cloutier et al., 2012; Cloutier & Gyurovski, 2013, 2014; Feng et al., 2016; Harris & Fiske, 2007; Hu et al., 2016; Le Bouc & Pessiglione, 2013; Ly et al., 2011; Meshi et al., 2013), nine articles reported foci based on social rank, which involved placing players and opponents in positions of high and low rank depending on task outcomes (Cikara et al., 2011; Kishida et al., 2012; Kumaran et al., 2012, 2016; Ligneul et al., 2016, 2017; Lindner et al., 2015; Op de Macks et al., 2017; Zink et al., 2008), two studies requested players to rate dominant/submissive faces (Chiao et al., 2008), or dominant/submissive bodily postures (Freeman et al., 2009), and two studies requested participants to rate profiles based on the player's similar or different personality traits (Beer & Hughes, 2010; Takahashi et al., 2009). From the 39 studies compiled for the monetary social comparison meta‐analysis, the majority of studies used the Ultimatum game (22 studies; see Tables 1 and 2 for details). Other common tasks used were the dot detection task in which the subject's rewards were compared with an opponents after estimating the number of dots on the screen (Du et al., 2013; Fliessbach et al., 2012), the bidding task, in which subjects competed with a computer to place bids on items to win money (Delgado et al., 2008; van den Bos et al., 2013), or game‐of‐chance task in which players compete with opponents to select one of three cards, each card containing a specific value (Dvash et al., 2010; Kang et al., 2013). See Tables 1 and 2 for a list of all studies with the corresponding stimuli and task type included in the analysis.

### 3.2 ES‐SDM maps

We analyzed upward and downward comparisons for both status and monetary contexts. A total of 20 and 13 contrasts were found for upward and downward social status comparisons, respectively. For the monetary comparisons meta‐analyses, 29 upward contrasts and 21 downward contrasts were included. It is important to emphasize that although the number of contrasts is deemed ineligible to conduct meta‐analyses using GingerALE software (Eickhoff, Laird, Fox, Lancaster, & Fox, 2017), ES‐SDM software utilizes the t score to improve statistical power, and validation of observed results is cross‐checked using the jackknife sensitivity analysis. Thus, a conservative minimum of 80% of studies was applied to validate which clusters were deemed replicable.

**FIGURE 2:**
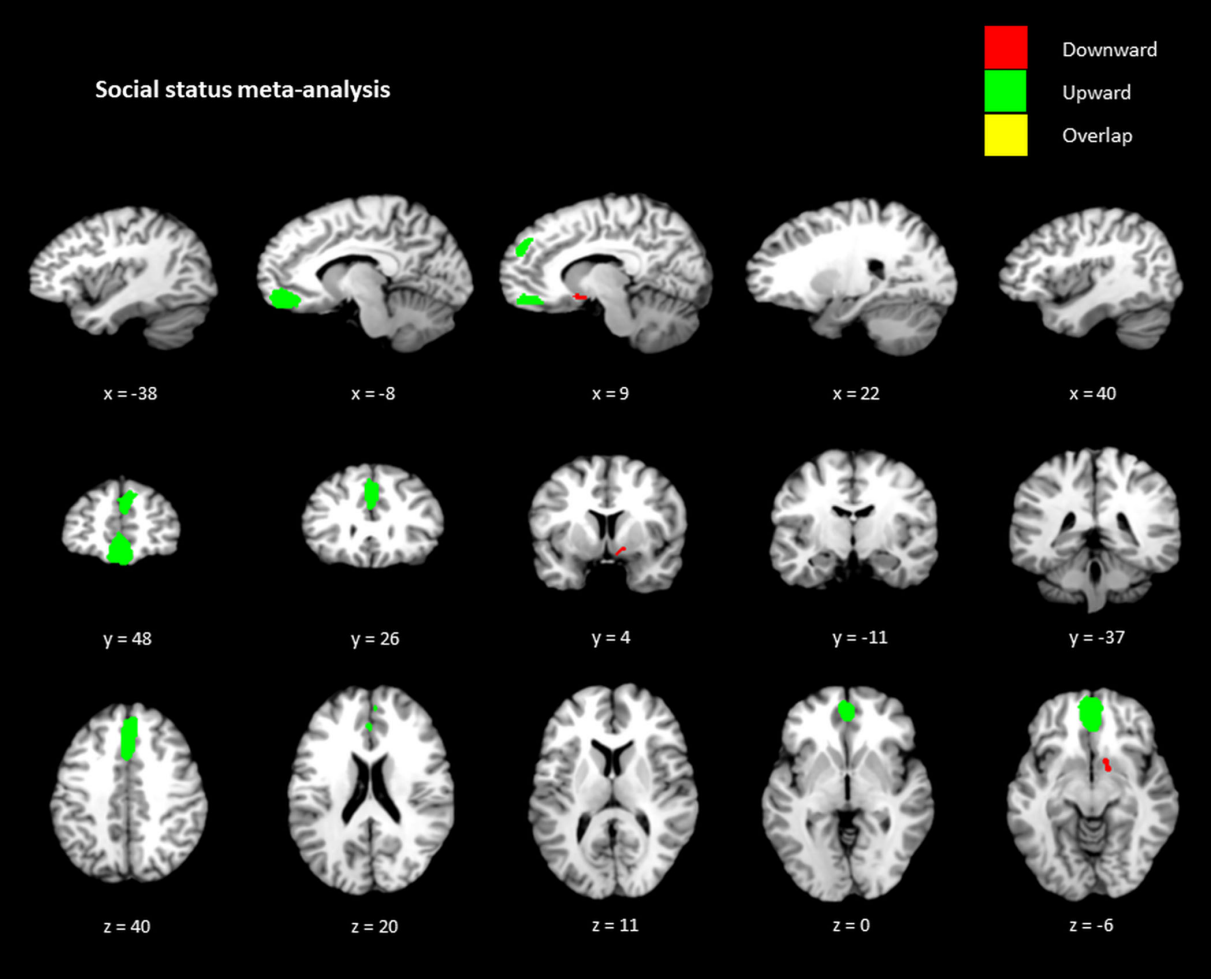
Monetary upward and downward comparisons

**FIGURE 3:**
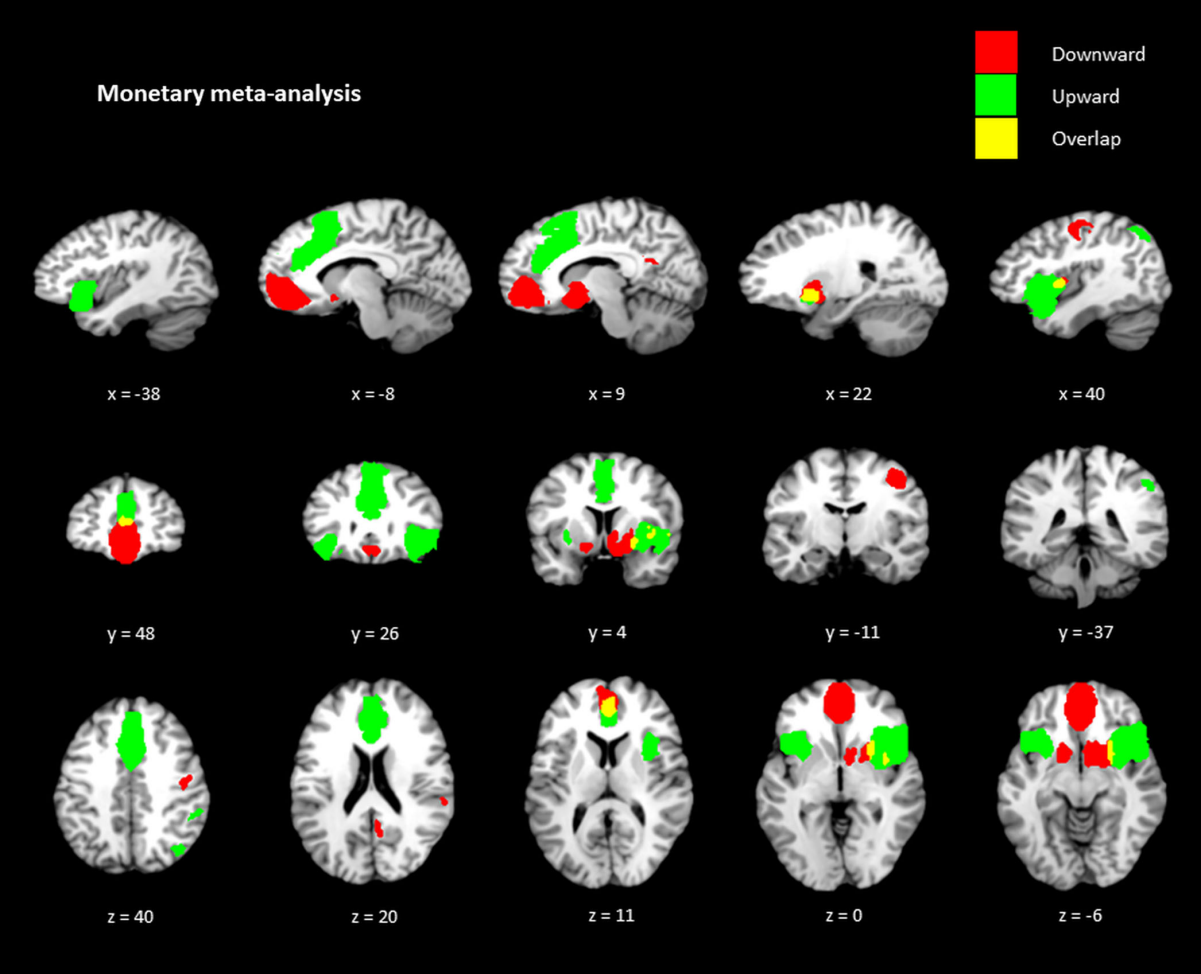
Social status upward and downward comparisons

For downward monetary comparisons (see Figure 2), a large cluster was reported within the OFC/ventral ACC (Brodmann Area [BA 10]), followed by a cluster within the right striatum, and right precentral gyrus (BA 6), and left striatum. Upward monetary comparisons revealed activity within the dorsal ACC (BA 32), right and left insula (BA 48), right angular gyrus (BA 7), and right supramarginal gyrus (BA 40). For social status downward comparisons (see Figure 3), the analysis reported one cluster reported within the right striatum. For social status upward comparisons, the analysis demonstrated a replicable cluster within the orbital frontal cortex/ventral ACC (BA 11) and a cluster within the dorsomedial PFC (BA 10/32). These clusters were deemed eligible according to the jackknife sensitivity analysis. The results of all areas for downward and upward social status and monetary comparisons are displayed in Table 3.

**TABLE 3:** Significant regions of activation for monetary comparisons split across upward and downward directions and significant regions of activation for social status comparisons split across upward and downward directions

Region	BA	*x*	*y*	*z*	SDM‐Z	*p*	Voxels^a^	Jackknife
**Significant regions of activation for monetary comparisons split across upward and downward directions**								
*Downward*								
Orbitofrontal gyrus	11	0	52	0	4.034	<.01e^−10^	2,288	100^b^
R Striatum		8	4	−4	3.610	8.762e^−6^	714	100^b^
R Precentral gyrus	6	44	−12	46	3.634	6.735e^−6^	248	95.4^b^
L Striatum		−14	12	−10	3.347	4.905e^−5^	164	100^b^
*Upward*								
Dorsal ACC	32	0	22	30	6.089	<.01e^−10^	4,829	100^b^
R Insula	48	32	16	0	6.064	<.01e^−10^	3,097	100^b^
L Insula	48(47)	−32	12	−6	5.114	<.01e^−10^	1,364	100^b^
R Angular gyrus	7(39)	38	−62	48	3.946	<.01e^−10^	196	100^b^
R Supramarginal gyrus	40	52	−38	42	3.601	1.239e^−5^	123	96.5^b^
**Significant regions of activation for social status comparisons split across upward and downward directions**								
*Downward*								
R striatum		12	2	−6	2.480	3.163e^−4^	22	83.3^b^
*Upward*								
Orbitofrontal gyrus	11	−2	44	−10	2.823	2.634e^−5^	956	100^b^
Dorsomedial PFC/dorsal ACC	10/32	4	36	36	2.964	8.762e^−6^	879	100^b^

*Note:* Jackknife replicability is represented as percentage; foci represented in MNI space; thresholded at *p* < .0005; FWHM 20 mm.

Abbreviations: ACC, anterior cingulate cortex; BA, Brodmann area; L, Left; PFC, prefrontal cortex; R, Right; SDM‐Z, signed differential mapping *z*‐score.

^a^ 2 mm × 2 mm × 2 mm.

b Regions greater than 80% replicability.

**FIGURE 4:**
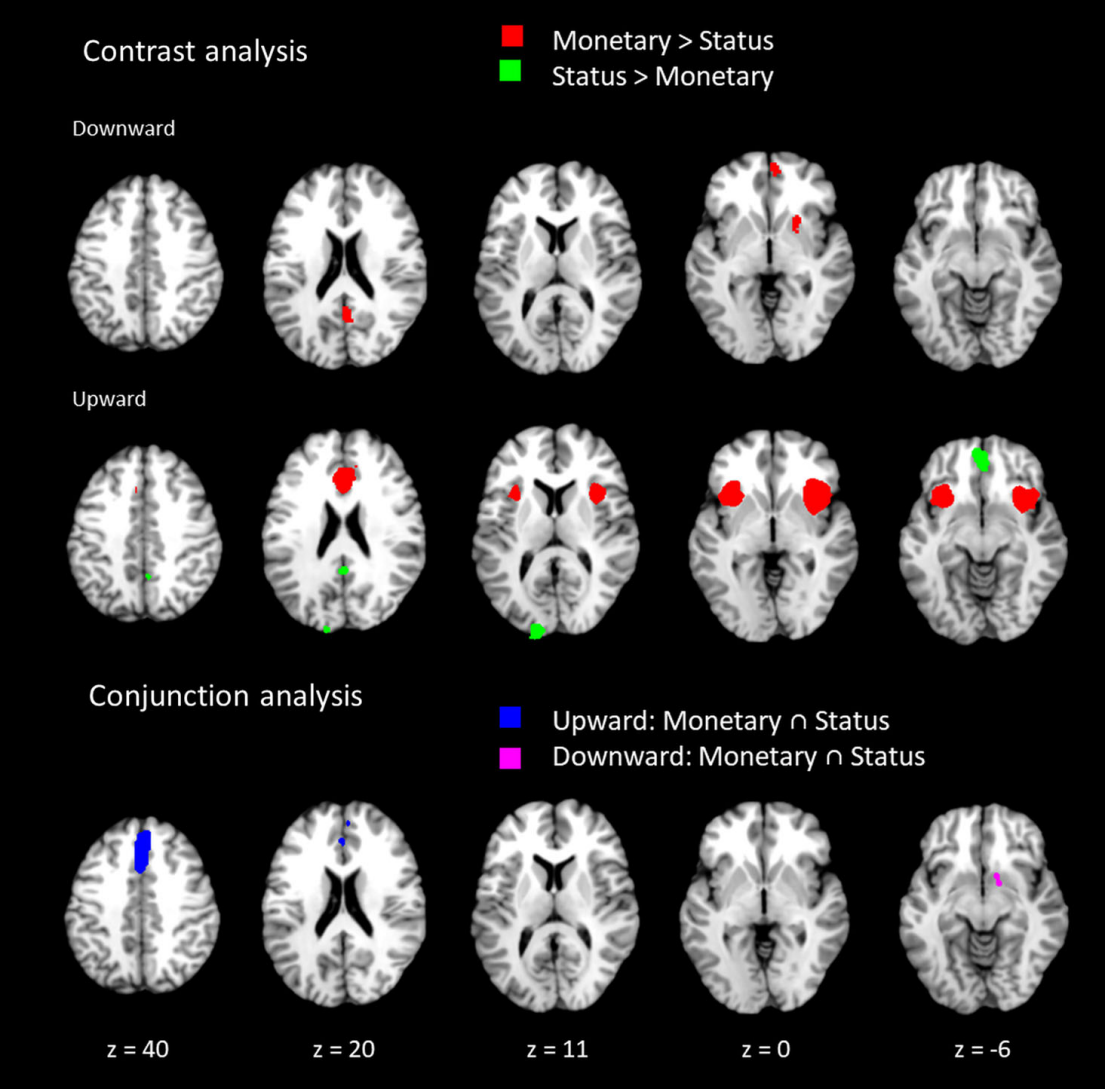
Contrast and conjunction analysis of monetary and status social comparisons

Interestingly, for both social status and monetary domains, meta‐analyses yielded a cluster within the right striatum for downward comparisons and a cluster in the dorsomedial PFC for upward comparisons, evident by the conjunction analysis (Figure 4). The results of the conjunction analysis of status and monetary split for upward and downward directions are displayed in Table 4. When comparing monetary with social status downward comparisons, monetary comparisons selectively recruited the OFC/ventral ACC, right striatum, right precuneus and right precentral gyrus as shown in the contrast analysis (Table 5). For upward comparisons, monetary comparisons selectively engaged bilateral insula and dorsal ACC, while social status comparisons exclusively recruited OFC/ventral ACC, left superior occipital gyrus and posterior cingulate gyrus (see Table 6).

**TABLE 4:** Conjunction analysis of status and monetary split for upward and downward directions

Region	BA	*x*	*y*	*z*	Voxels^a^
*Downward*					
R Striatum		12	2	−6	22
*Upward*					
Dorsomedial PFC	10/32	4	36	36	804

*Note:* Foci represented in MNI space; thresholded at *p* < .0005; FWHM 20 mm.

Abbreviations: BA, Brodmann area; PFC, prefrontal cortex; R, Right.

a 2 mm × 2 mm × 2 mm.

**TABLE 5:** Contrast analysis of status and monetary split for downward directions

Region	BA	*x*	*y*	*z*	SDM‐Z	*p*	Voxels^a^
*Monetary > Status*							
Orbitofrontal gyrus	10	4	52	6	1.771	2.028e^−4^	74
R Striatum		22	10	2	1.828	1.295e^−4^	40
R Precuneus	23	4	−54	22	1.757	2.219e^−4^	24
R Precentral gyrus	6	38	−14	46	1.763	2.126e^−4^	23
*Status > Monetary*							
No suprathreshold clusters							

*Note:* Foci represented in MNI space; thresholded at *p* < .0005; FWHM 20 mm.

Abbreviations: BA, Brodmann area; R, Right; SDM‐Z, signed differential mapping *z*‐score.

a 2 mm × 2 mm × 2 mm.

**TABLE 6:** Contrast analysis of status and monetary split for upward directions

Region	BA	*x*	*y*	*z*	SDM‐Z	*p*	Voxels^a^
*Monetary > Status*							
R Insula	48	34	16	4	3.839	<.01e^−10^	1,197
L Insula	48	−30	16	−6	3.327	<.01e^−10^	817
Dorsal ACC	24/32	4	22	24	2.509	5.662e^−6^	699
*Status > Monetary*							
Orbitofrontal gyrus	11	2	44	−14	2.506	1.859e^−5^	771
L Superior Occipital gyrus	17	−14	−96	18	2.351	3.713e^−5^	279
Posterior Cingulate gyrus	23	0	−44	28	2.075	1.367e^−4^	290

*Note:* Foci represented in MNI space; thresholded at *p* < .0005; FWHM 20 mm.

Abbreviations: ACC, anterior cingulate cortex; BA, Brodmann area; L, Left; R, Right; SDM‐Z, signed differential mapping *z*‐score.

a 2 mm × 2 mm × 2 mm.

## 4 DISCUSSION

For this study we were determined to test the common currency hypothesis for social status and monetary comparisons by performing a series of fMRI meta‐analyses. Common‐currency hypothesis assumes that the brain uses a ‘common currency’ to rank outcomes and actions analogous to monetary gains and losses (Landreth & Bickle, 2008). According to this hypothesis, we expected to show a similar pattern of activity for monetary and social status. First we adopted social comparison theory to operationally define downward and upward comparisons. To test the common‐currency hypothesis for monetary and social status contexts, we expected both brain maps to yield regions associated with reward and losses. This assumption was based on a previous meta‐analysis (Luo et al., 2018) showing that downward comparisons recruit reward‐related striatum while upward comparisons yielded bilateral insula and anterior cingulate cortical activation, reflecting neural processes associated with losses. Due to the inclusion of monetary and status‐related contrasts in this prior report, we found it necessary to separately perform and contrast meta‐analyses of monetary and status upward and downward comparisons.

### 4.1 Common activity for downward and upward comparisons

First and foremost, meta‐analyses of downward comparisons in both domains demonstrated common patterns of activity, namely activity within the right striatum. The right striatum is associated with learning associations between rewarding stimuli and motor responses (Pizzagalli, 2014), habitual learning (Patterson & Knowlton, 2018) and with learning new stimulus–reward contingencies (Knutson & Cooper, 2005; Rogers et al., 2004). This result confirms the notion that downward comparisons in both monetary and status domains are compatible with the common currency hypothesis since the striatum is typically associated with the reception of reward (Delgado, 2007; Haber & Knutson, 2010).

The common currency hypothesis would also predict regions associated with losses during upward comparisons such as the ACC and insula (Luo et al., 2018). Indeed, our meta‐analyses of upward comparisons in both domains demonstrated common patterns of activity, namely activity within the dorsal ACC (dACC). For status and monetary upward comparisons, the dorsal ACC was concordant across studies. Among social comparisons, the ACC is often associated with psychosocial functioning such as social neglect (Lockwood, Apps, Roiser, & Viding, 2015; Lockwood & Wittmann, 2018; van der Molen, Dekkers, Westenberg, van der Veen, & van der Molen, 2017), monitoring of other people's decisions (Apps, Balsters, & Ramnani, 2012), and motivated social cognition (Hughes & Beer, 2012; Wittmann, Lockwood, & Rushworth, 2018). This may suggest that the ACC is a common active region associated with social interactions. The dorsal ACC also plays a key role in the processing of prediction errors and expectation violation (Kedia et al., 2014; van der Molen et al., 2017), which may corroborate the common currency hypothesis since individuals viewing others as beneficial may reflect a “worse than expected” prediction error (van der Molen et al., 2017; Yu & Zhang, 2014).

Such downward‐striatum and upward‐dACC activity patterns in social comparison across domains suggest that the basic reward and aversion brain systems are underlying this well‐known social phenomenon, regardless of the social settings. These findings highlight the pervasiveness of human tendency to compare with others and point out that such tendency is closed linked to the basic reward evaluation system. Our study may help explain why humans are prone to social comparison in all types of social areas, ranging from important social dimensions like attractiveness, wealth, and intelligence, to trivial things such as speech order and seating arrangement. It has been demonstrated that humans learn and evaluate values in a relative—context‐dependent—scale such that the context value sets the reference point to which an outcome should be compared (Palminteri, Khamassi, Joffily, & Coricelli, 2015). Hence, individuals may drive pleasure for winning $100 in the context of others winning only $10 or for publishing a paper in a mediocre journal in the context of colleagues having no publications. The reward evaluation system may convert all values to a common currency and scale it to a relative value so that even a small value can have huge impact on an individual's emotions. The computational mechanisms of common currency evaluation circuits may help explain how individuals respond to social comparison in different domains and with different magnitude of importance.

### 4.2 Unique activity for monetary and status comparisons

The contrast analyses revealed domain‐specific activity in social status and monetary comparisons. For example, downward comparisons in the monetary domain recruited greater activity within the orbital frontal gyrus/ventral ACC, right striatum as well as right precuneus and precentral gyrus, while status downward comparisons revealed no additional clusters, indicating additional processes for monetary compared to status comparisons. Upward monetary comparisons demonstrated larger clusters within the bilateral insula and dorsal anterior cingulate cortex compared to status comparisons. The insula may account for evaluations of social comparisons since the insula has been attributed to anticipating and evaluating the consequences of one's actions (Simmons et al., 2011; Späti et al., 2014), and self‐initiated actions in social exclusion trials (Wang et al., 2019). Additional activity in monetary domain for downward comparison may indicate that financial advantage is more salient than social status advantage. On the other hand, social status upward comparisons yielded activity within the orbitofrontal cortex/ventral ACC, as well as left superior occipital and posterior cingulate cortex. Such unique activity pattern for social upward comparison may speak to the strong motivational nature of lagging behind in social ladders. Being lower in social status is an important teaching signal for individuals as it is often linked to social defeat and other disadvantages when acquiring social resources. Lower social ranking in the animal kingdom may also be associated with social threat and being intimidated by higher ranking others. Interestingly, the orbital frontal cortex was the only region active in both upward and downward comparisons, greater in the monetary context when making downward comparisons, yet greater in the social status context when comparing others with higher status. The orbital frontal cortex is an area commonly known for evaluating value of goals and decisions (Elliott, Newman, Longe, & Deakin, 2003; Hare, O'Doherty, Camerer, Schultz, & Rangel, 2008; O'Doherty, Critchley, Deichmann, & Dolan, 2003), and which has been implicated in both lesion (Karafin et al., 2004; Mah et al., 2004) and empirical fMRI studies investigating social hierarchy (Kumaran et al., 2016). Recently a large meta‐analysis had shown that the medial orbital part of the medial prefrontal cortex (i.e., the ventral medial prefrontal cortex; BA 11) is specifically recruited for the processing of situations, while processing of self and others recruits mainly the anteromedial and dorsomedial sub‐regions of the prefrontal cortex, respectively (Lieberman, Straccia, Meyer, Du, & Tan, 2019). With regard to the current study, this may suggest that upward status and downward monetary comparisons involve situational processing since both yielded medial prefrontal cortical activity (labeled as orbitofrontal cortex in Tables 3, 4, 5, and 6). However, downward monetary comparisons appeared to yield a medial prefrontal cortex cluster slightly more anterior than the upward status comparison contrast, possibly indicating additional self‐referential processing.

Upward and downward social comparisons have been shown to reflect both positive and negative outcomes. For instance, evaluation of others in the upward direction may include admiration or envy toward superior peers (Hagerty, 2000; Suls, Martin, & Wheeler, 2002) whereas downward comparisons may lead to the encouragement of subordinates to strive for success rather than gloat over one's own gains (Gibbons, 1986; Wills, 1981). The finding that the OFC is activated by both upward and downward comparison may suggest that humans are actively engaged in the detection of “self‐other” differences. Monitoring whether others are different from us, regardless of being better or worse, may help mobilize resources to evaluate and resolve the social deviation. This finding indicates that social comparison is an important learning process that helps individuals to sense whether anything, in comparison with others, is out of order. Perhaps differential activation of the orbitofrontal cortex may relate to how one may be evaluating others since this region is the only region to be associated with both directions of the comparison and is functionally related to evaluation (Cloutier & Gyurovski, 2014; Elliott et al., 2003; Hare et al., 2008; O'Doherty et al., 2003). However, this notion has yet to be tested in an empirical setting. Moreover, we were unable to distinguish upward and downward comparisons that were either worse or better than expected. Few articles examined whether participants perform better or worse than someone lower in the social hierarchy (Zink et al., 2008), which may account for the functional differences in upward and downward comparisons. Potentially achieving a higher superior position could be a rewarding experience but also be associated with antagonistic retaliation (Fiske, 2010). The interaction between social status and relative performance and its relationship to the orbitofrontal cortex could be an exciting topic for future neuroimaging research.

## 5 CONCLUSION

To sum, brain areas most likely to be active in an fMRI study investigating monetary and status comparisons seem to dovetail with regions associated with the reward system (the striatum), regions associated with monetary losses (ACC and bilateral insula). These meta‐analyses suggest that there are overlapping as well as distinct brain networks associated with social comparisons in monetary and social status domains. Our meta‐analysis results, however, should be interpreted with caution due to potential reverse inference. Future empirical research may directly compare social comparisons in different domains in the same study. Nevertheless, our findings and interpretations may inform current models on social comparisons in competitive social settings and improve our understanding of the neural mechanisms that are associated with these behaviors.

## Data Availability

Data sharing is not applicable to this article as no new data were created or analyzed in this study.
